# The Pupil in Its Semeiological Aspects

**Published:** 1887-08

**Authors:** 


### THE PUPIL IN ITS SEMEIOLOGICAL ASPECTS.

In a very interesting paper by Macewan, in the *American Journal of the Medical Sciences* for July, 1887, the following conclusions are presented:

(A) 1. When the function of the brain is in abeyance, the pupils are in a state of stabile mydriasis.

2. This may arise either from temporary suspension or from aboliton of function.

3. Temporal suspension is illustrated by shock, and the effect of some poisons; while the abolition of function is exemplified by extensive laceration and compression of the brain.

(B) 4. When the function of the brain is interfered with by conditions usually included under the term "irritation," the pupils are in a state of myosis; sometimes labile, but generally stabile myosis.

5. This "irritation" or interruption of function may be seen during certain degress of cerebral anæmia, produced experimentally, and not a pathological result; certain amounts of brain pressure, and certain stages of intracranial inflammation.

6. These are illustrated in persons who have suddenly lost a considerable quantity of blood (about a fifth of the whole); in the growth of intracranial tumors, and the formation of sanguinolent serous and purulent effusions, when the degree of pressure may be denominated as "medium," and at certain periods of meningitis and encephalitis.

(C) 7. The same pathological factors which cause myosis may also cause mydriasis, the degree in which these factors are present being the determining point between the former and the latter, and not *merely* the particular locus in the brain.

8. It is well illustrated by cases where the hemorrhage is repeated, and is finally pushed to syncope; in intracranial pressure, which is gradually increased until it becomes great, such as arises from tumors, blood-clots, and inflammatory products.

(D) 9. When the function of one-half of the cerebrum is placed in abeyance by a superficial or cortical lesion, the pupil on the same side as the lesion is in a stabile mydriasis.

10. This is well illustrated in cases of intracranial sanguinolent effusion consequent on injury (see list of cases).

(E) 11. When the function of one-half of the cerebrum is interfered with by some source of cortical irritation, the pupil on the corresponding side to the lesion is in a state of myosis.

12. This is illustrated by traumatic and pathological lesions affecting the cortex of the cerebrum.

(F) 13. Hemorrhage into the pons Varolii when small, causes strongly contracted pupils ; but when it is more extensive, involving the gray matter beneath the aqueduct of Sylvius, a state of stabile mydriasis is induced.

14. Effusions into the lateral ventricles when small, produce contraction of the pupils, but when the effusion is great stabile mydriasis ensues.

15. Inequality of the pupils indicates a unilateral lesion or lesions.

16. When the lesion is cortical and unilateral the pupillary manifestations are on the corresponding side. When the basal nerves are affected unilaterally the pupillary effect is manifested on the same side as the lesion. When the lesion is unilateral and affects the function of the white fibres of the cerebrum, the opposite pupil is generally affected. When the basal ganglia are implicated unilaterally the pupil is sometimes affected on the same side as the lesion, occasionally on the other side.

In a case of cholesteatoma and in another of glioma of the right optic thalamus, dilatation of the left pupil was found (*Ross*, vol. ii., p. 572).

In lesions of the cerebral peduncles the pupil is affected on the same side as the lesion.

Lesions in the corpora quadrigemina affect both pupils, irritation* causing contraction, destruction causing dilatation and immobility.

Section or destruction of one optic tract causes dilatation of the opposite pupil and blindness of the opposite eye.

17. Irritation of the cord, especially the cilio-spinal axis, produces dilatation of the pupils, while destruction of the cord causes contraction. These effects are generally seen in both pupils, though, experimentally at least, they may be confined to the same side as the lesion.

18. The pupils are affected in the same way by lesions of the sympathetic, though in unilateral lesions it is only the pupil on the same side as the lesion which is affected.

19. Speaking generally, when myosis is due to a cerebral cause, it indicates the earlier stages of various affections ; when due to a spinal lesion it points to a most serious paralysis, often to the destruction of the part. When mydriasis arises from a cerebral lesion it is generally present in large amount ; when due to a spinal affection it indicates irritation of the part.

Myosis Occurs under the Following Conditions :

1. When a bright light acts upon the retina.
2. Accommodation for a near object.
3. Rotation of the eyeball inward.
4. Local irritation or painful affections of the eyeball.
5. Irritation of the oculomotor nerve.
6. Paralysis of sympathetic roots of lenticular ganglion or trunk of sympathetic in the neck. In paralysis of the fifth there is myosis and inflammation passing on to destruction of the eyeball.
7. Paralysis of the cilio-spinal region of spinal cord. All affections which destroy the cervical spinal marrow and intercept its conductibility produce congestion of the face and contraction of the pupils. In neurosis, which suspends or diminishes the tone of the sympathetic or spinal axis.
8. Encephalic congestion, such as : obstacle to return of blood in jugulars ; venous congestion due to cardiac causes ; active hyperæmia, plethora, fevers, pneumonia, hepatitis, etc. ; when animal is suspended by the heels ; in early stages of meningitis and encephalitis ; in acute mania with marked activity of the cerebral circulation ; in chronic mania pupils are variable, when contracted are said to

*Unilateral electrical stimulation causes dilatation of both pupils, the opposite one becoming first dilated. Ferrier explains this as the result of irritation of a sensory structure, in this case acting through the medium of the anterior roots of the second dorsal nerves which ascend in the cervical sympathetic.

indicate supervention of paralytic dementia.

9. During sleep; some believe this to be due to the congestion of the cerebral vessels and those of the iris (Mosso); others, to the inward rotation of the eyeball.

10. In early stages of cerebral tumor.

11. In small hemorrhages into the cerebellum. In irritation of the cerebellum, contraction of the pupil on same side as lesion ensues.

12. Electrical stimulation of the angular gyrus frequently causes contraction of the pupil.

13. During forced expiration, when the eye is at the same time passive. Also generally seen during the period of apnœa in Cheyne-Stokes respiration.

14. Convulsions arising from meningo-encephalitis are said to be accompanied by myosis, while in convulsions due to epilepsy and in epileptiform fits they are usually accompanied by mydriasis.

15. When the eye contracts on accommodation to a near object, yet does not contract to light, this indicates a lesion situated between the corpora quadrigemina and oculomotorius. This affection is known as the Argyle-Robertson symptom. It is seen in locomotor ataxia, and occurs in the progressive paralysis of the insane.

16. During uræmic coma.

17. Myotics: physostigmine, nicotine, pilocarpine, morphine, muscarine.

MYDRIASIS OCCURS UNDER THE FOLLOWING CONDITIONS:

1. In darkness or in subdued light.

2. Accommodation for distant objects.

3. Rotation of the eyeball outward.

4. In forced movements discharged from the medulla; vomiting, swallowing, chewing, forced respiration.

5. Paralysis of the oculomotor (accompanied or not by immobility of eyeball, external strabismus, diplopia, etc.).

6. Destruction of the optic nerve; amaurosis. When unilateral, associated movements continue.

7. Irritation of sympathetic; powerful impressions on sensory nerves; strong moral emotions, mental pain, grief, fear; neuralgia of the fifth nerve.

8. Irritation of the spinal cord, especially cilio-spinal region.

9. Encephalic anæmia: In all cases where there is reflex contraction of the vessels of the head; when loss of blood from the body is excessive; obstruction of the carotid arteries; in thrombosis of brain sinuses; dilatation of mesenteric vessels when extreme; syncope, intense cold, rigors; dyscrasias of the blood, convalescence, cachectic conditions; asphyxia, epilepsy, in certain stages of these affections.

10. Pressure of cerebrum, when great in amount, as from hemorrhage, neoplasms, etc. In the last stages of meningo-encephalitis.

11. In cerebral softening. In acute dementia (œdema of cortex cerebri) observers state that the pupils are invariably dilated (Hutchinson).

12. In idiots the pupils are generally dilated.

13. During deep inspiration, generally in respiratory period of Cheyne-Stokes breathing.

14. Hemorrhage into centrum ovale and into cerebral peduncles.

15. Ferrier produced dilatation of opposite pupil by destructive lesion of the optic tract in the thalamus, indicative of rupture of the centripetal fibres to the irido-motor nucleus in the floor of the Sylvian aqueduct.

16. In hydrophobia there is mydriasis.

17. Mydriatics: atropine, homatropine, duboisine, daturine, hyoscyamine. Curare injected subcutaneously in animals (five to ten centigrammes) induces in one or two hours complete paralysis of the third nerve.

THE EFFECT ON THE PUPIL OF LOCAL CONDITIONS OF THE EYEBALL:*

1. Hyperæmia of the iris produces contraction of the pupil which darkness scarcely diminishes.

2. Presbyopia and hypermetropia

* The writer is indebted to Dr. Thomas Reid for revising this list of pulpillary effect occasioned by local conditions of the eyeball.

cause contraction of the pupils in cases where continuous and excessive strain for near accommodation has been long continued, and has produced asthenopia.

3. Pupillary atresia, consequent upon chronic irritation with posterior synechia, producing contraction of the pupil.

4. In synechia total dilatation is impossible, the iris dilating only where free, hence the pupil is irregular. If the synechia is annular the pupil is both contracted and immobile.

5. In microria there is a congenital state of extreme contraction.

6. In glaucoma the pupil is dilated, contracting little or not all to the action of calabar bean.

7. In coloboma, both in the congenital form and after iridectomy, there are irregularity and immobility of the pupil.

8. In idiopathic mydriasis there is little contraction to the action of light or myotics.

9. In certain cases of amblyopia and amaurosis there is no dilatation of the pupil.

10. In hippus pupillæ there are alternate contraction and dilatation often accompanied by nystagmus.

11. Inequality of the pupils exists in some who have different degrees of refraction in the two eyes, one being emmetropic and the other myopic.—*Quarterly Compendium of Medical Science.*

